# Supplementary material for: Impact of tissue staining and scanner variation on the performance of pathology foundation models: a study of sarcomas and their mimics
Source: J Pathol Clin Res. 2026 Feb 24;12(2):e70080. doi: 10.1002/2056-4538.70080 (PMC12932120; doi:10.1002/2056-4538.70080)
Supplement: Supplementary file 1 — Figure S1. Principal component analysis (PCA) of stain vectors extracted from each 3,000 × 3,000 pixel region of interest, showing the plot by each scanner Figure S2. Serial sections from a sample of a leiomyosarcoma stained at 30 different NHS Trusts show significant colour variation after digitisation with the same Aperio scanner Figure S3. Visualisation of the feature space using t‐SNE Table S1. Case IDs and diagnoses for all cohorts Table S2. Cohort 4 case distribution Table S3. Top‐1 and top‐3 classification accuracies on Cohort 4‐Test set for ‘trained with Cohort 1’ and ‘trained with the Combined cohort’ Table S4. Class‐wise accuracies for soft tissue tumours across internal and multi‐institutional test sets, using seven models [file CJP2-12-e70080-s001.zip › cjp270080-sup-0001-FiguresS1-S3TablesS2-S4.pdf]

Impact of tissue staining and scanner variation on the performance of pathology foundation models: a study of sarcomas and their mimics

B Chai, J Chen *et al*. *J Pathol Clin Res* https://doi.org/10.1002/2056-4538.70080

Supplementary Figures S1–S3

Supplementary Tables S2–S4. Table S1 is provided as a separate Excel file.

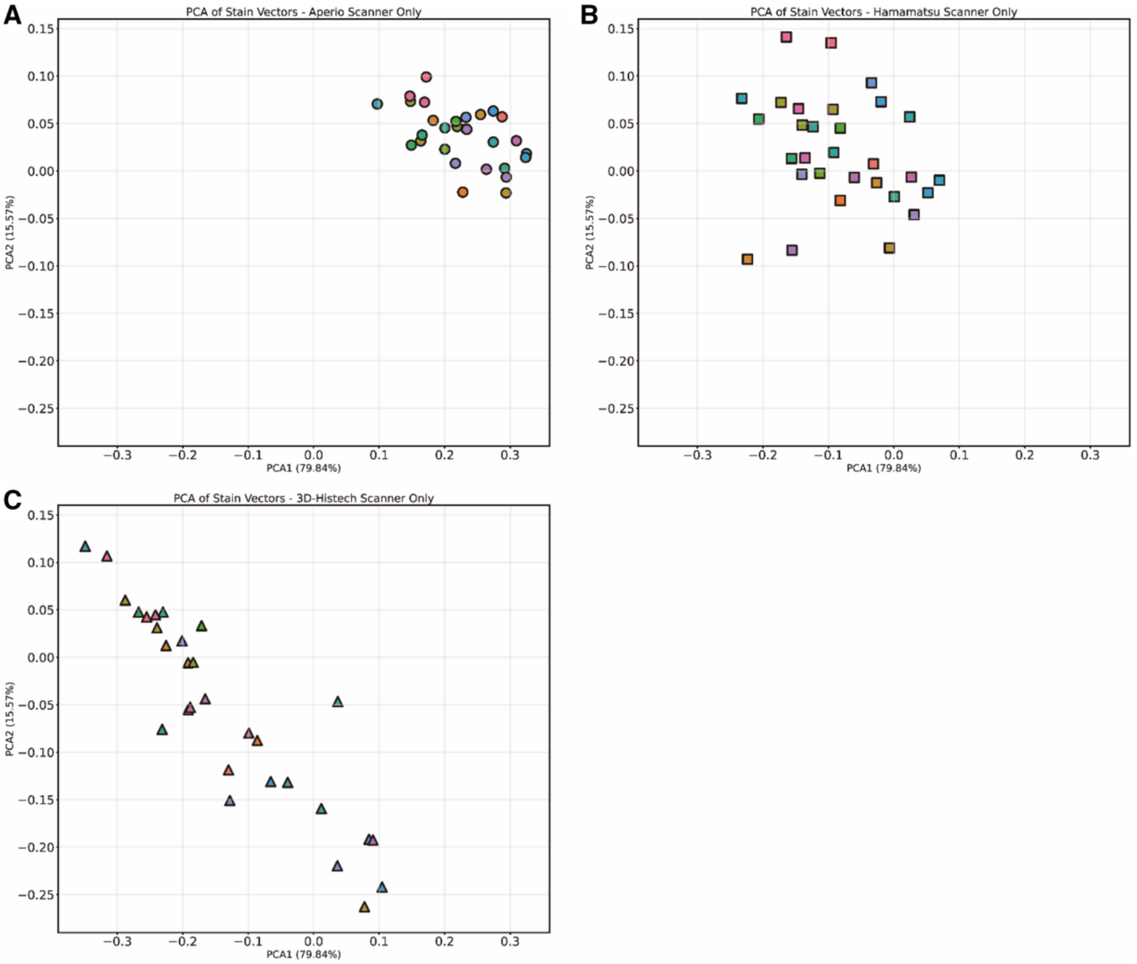

Figure S1. Principal component analysis (PCA) of stain vectors extracted from each $3{,}000 \times 3{,}000$ pixel region of interest, showing the plot by each scanner: (A) Aperio, (B) Hamamatsu, and (C) 3D-Histech.

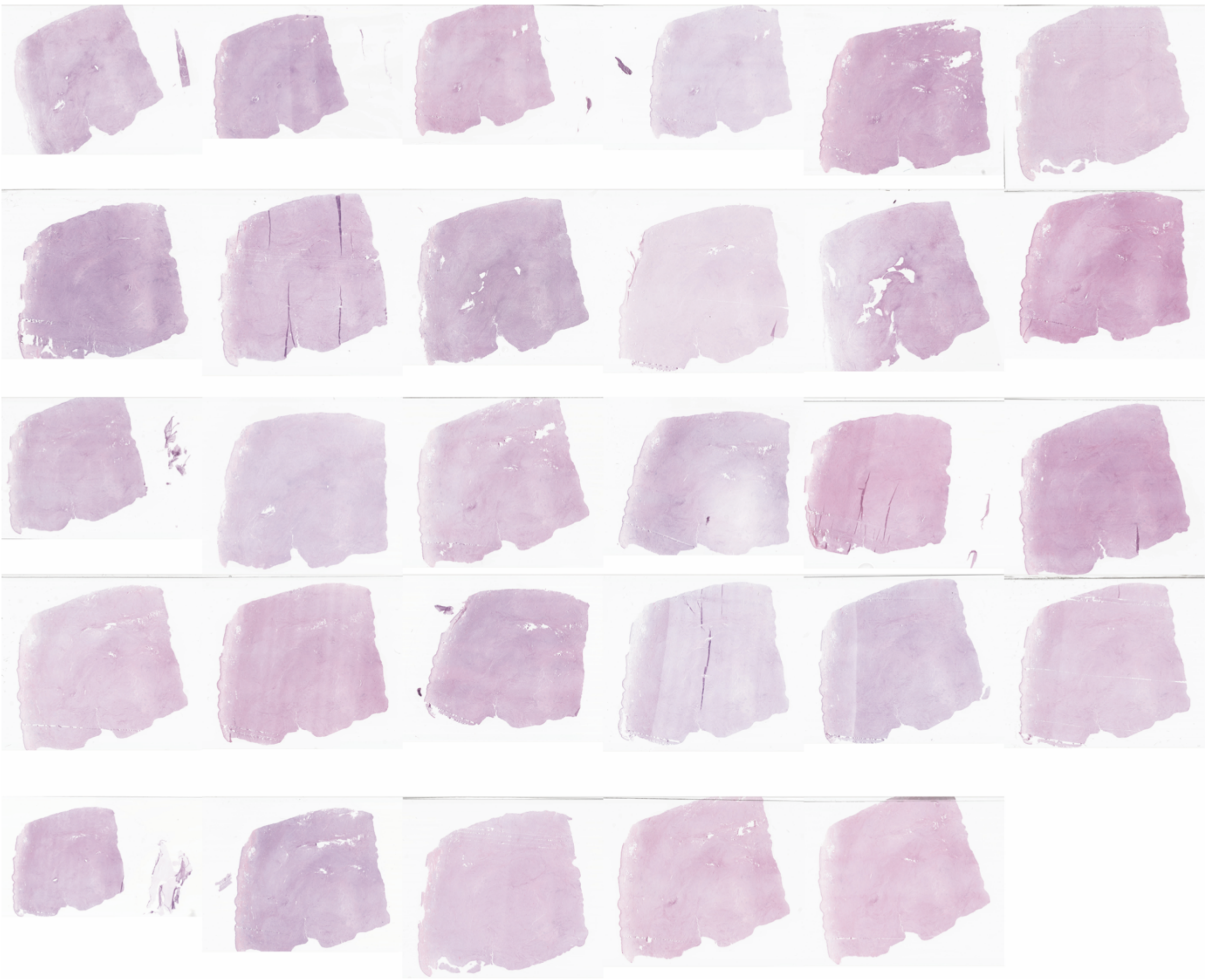

Figure S2. Serial sections from a sample of a leiomyosarcoma stained at 30 different NHS Trusts show significant colour variation after digitisation with the same Aperio scanner. One slide was not returned.

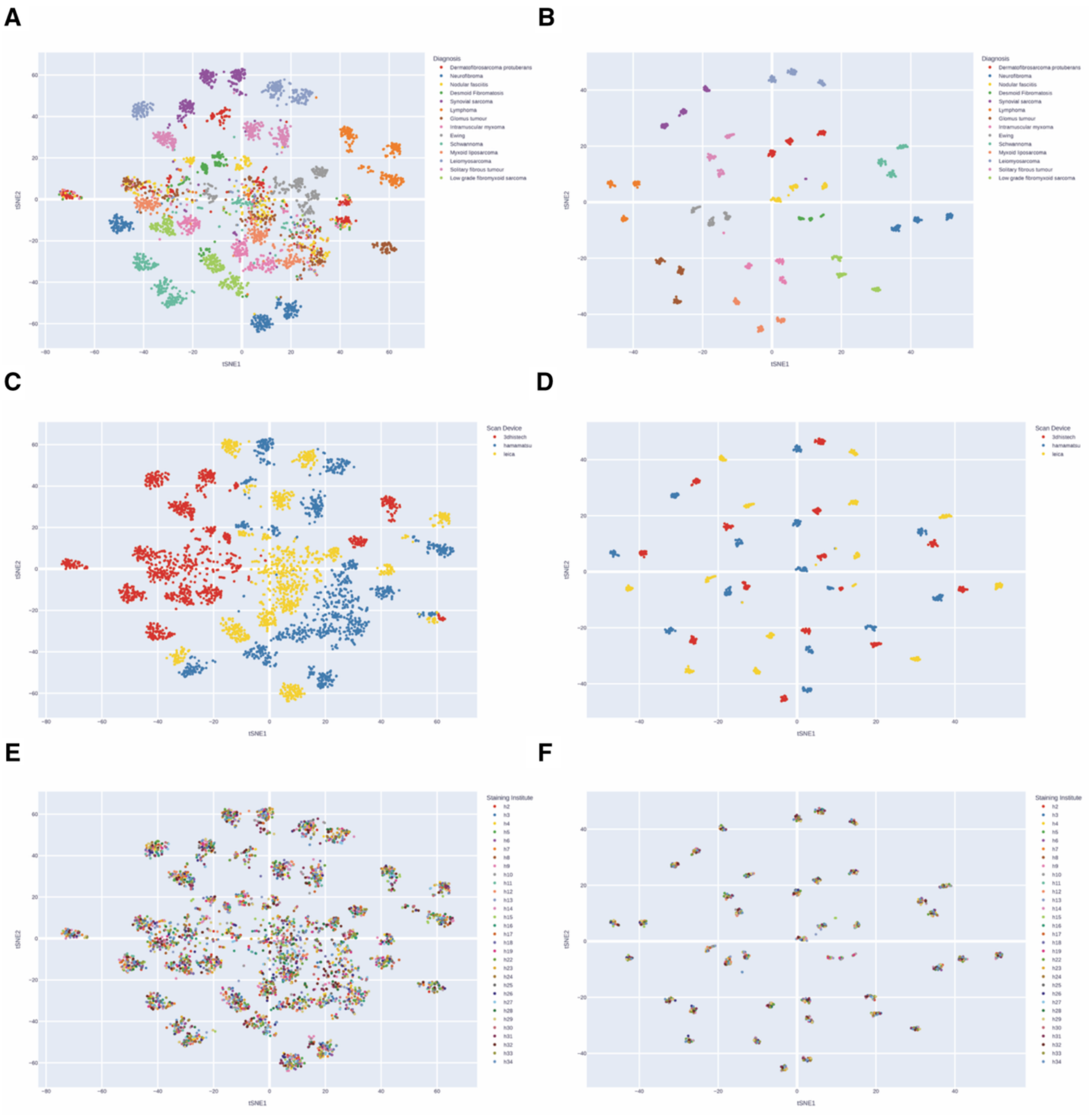

Figure S3. Visualisation of the feature space using t-SNE. (A, C, E) Tile-level embeddings from the UNI-v2 model for the staining- and scanning-controlled dataset (Cohort 2). For each slide, three tiles were randomly sampled, resulting in three points per slide in the visualisation. The embeddings are coloured by diagnosis (A), scan device (C), and staining institute (E), respectively. (B, D, F) Slide-level embeddings from the TITAN model for the same dataset, with one point per slide, coloured by diagnosis (B), scan device (D), and staining institute (F). An interactive version of these t-SNE plots is provided (see Data availability statement).

Table S1. See separate Excel file

Table S2. Cohort 4 case distribution

Staining Institute	Nr. Cases	Staining Institute	Nr. Cases
University College Hospital London	170	Royal Belfast Hospital	2
Basel	159	Royal Marsden Hospital	2
Royal National Orthopaedic Hospital	105	St James Hospital	2
The Robert Jones and Agnes Hunt Orthopaedic Hospital	97	Southmead Hospital	2
Queen Square	43	Royal London Hospital	2
St Vincent's University Hospital	28	Advance Histopathology Laboratory	2
DHM, Sydney	27	Broomfield Hospital	2
Southampton General Hospital	26	University Hospital of Wales	2
Instituto Valenciano de Oncologia	24	Royal Marsden Hospital	2
The Valencian Institute of Oncology, Spain	23	Overseas Hospital	2
Charing Cross Hospital	18	Queen's Hospital	2
North West London Pathology	16	Hillingdon Hospital	1
Royal Brompton Hospital	13	Mater Misericordiae University Hospital	1
Great Ormond Street Hospital	12	Portland Hospital	1
National Hospital for Neurology and Neurosurgery	11	Royal Infirmary of Edinburgh	1
Royal Free Hospital	10	Clemintine Churchill Hospital	1
The Royal London Hospital	10	Platinum Medical Centre	1
HCA Healthcare	8	King's College Hospital	1
Princess Alexandra Hospital	7	Queen Alexandra Hospital	1
Basildon and Thurrock University Hospitals	6	Royal Victoria Infirmary	1
University Hospital Southampton	6	Royal Bournemouth Hospital	1
Hemel Hempstead Hospital	5	County Durham and Darlington NHS Foundation Trust	1
Northwick Park Hospital	5	Barts Health NHS Trust	1
Queen's Hospital	5	St Thomas' Hospital	1
Ipswich Hospital	5	Colchester Hospital	1
Luton and Dunstable University Hospital	5	Queen Elizabeth Hospital Woolwich	1
Spire Bushey Hospital	5	Queen Victoria Hospital	1
St George's Hospital	4	Mount Vernon Hospital	1
Guy's Hospital	4	Antrim Area Hospital	1
Lister Hospital	4	Maidstone and Tunbridge Wells Hospital	1
Southend University Hospital	4	Aglaia Kyriakou Hospital	1
Nuffield Orthopaedic Centre	4	Royal Surrey County Hospital	1
Norfolk and Norwich University Hospital	4	West Herts Histology	1
Addenbrooke's Hospital	3	Hammersmith Hospital	1
Salisbury District Hospital	3	Ealing	1
Cork University Hospital	3	Cellular Pathology Services	1
Bon Secours Hospital	3	**Total cases**	**934**
Sheffield Teaching Hospitals	2	**Total staining institutes**	**74**

Table S3. Top-1 and top-3 classification accuracies on Cohort 4 test set for 'trained with Cohort 1' and 'trained with the Combined cohort'

	Google	UNI-v2	CONCH-v1.5	TITAN	Virchow	PRISM	CNN
**Top-1 (Cohort 1)**	0.721	0.847	0.772	0.860	0.852	0.782	0.533
**Top-1 (the Combined cohort)**	0.808	0.899	0.838	0.881	0.865	0.803	0.736
**Top-3 (Cohort 1)**	0.934	0.970	0.938	0.968	0.963	0.940	0.844
**Top-3 (the Combined cohort)**	0.946	0.983	0.970	0.970	0.967	0.958	0.923

Table S4. Class-wise accuracies for soft tissue tumours across internal and multi-institutional test sets, using seven models: Google Path Foundation, UNI-v2, CONCH-v1.5, TITAN, Virchow, PRISM, and ResNet50-CNN

**Trained with only internal set (Cohort 1 Train), tested on internal set (Cohort 1 Test)**							
**Subtype**	**Google**	**UNI-v2**	**CONCH-v1.5**	**TITAN**	**Virchow**	**PRISM**	**ResNet50**
Dermatofibrosarcoma protuberans	0.900	0.900	0.850	0.950	0.850	0.850	0.650
Neurofibroma	0.800	1.000	0.950	0.750	0.950	0.750	0.650
Nodular fasciitis	0.850	0.850	0.850	0.950	0.944	0.850	0.800
Desmoid fibromatosis	0.950	0.950	0.850	0.900	0.900	0.900	0.950
Synovial sarcoma	0.950	0.850	0.900	0.900	0.850	0.950	0.850
Lymphoma	0.850	1.000	1.000	1.000	1.000	1.000	0.850
Glomus tumour	1.000	1.000	1.000	1.000	1.000	1.000	0.900
Intramuscular myxoma	1.000	1.000	1.000	1.000	1.000	1.000	0.900
Ewing	0.850	1.000	0.950	0.950	1.000	0.950	0.900
Schwannoma	0.900	0.900	0.900	0.900	0.900	0.900	0.850
Myxoid liposarcoma	0.900	0.900	0.800	1.000	0.800	0.850	0.750
Leiomyosarcoma	0.850	0.850	0.850	0.850	0.850	0.700	0.850
Solitary fibrous tumour	0.950	0.950	0.900	0.950	0.900	0.800	0.750
Low grade fibromyxoid sarcoma	1.000	1.000	0.950	0.950	0.950	0.850	0.850
**Overall accuracy**	**0.911**	**0.939**	**0.911**	**0.932**	**0.921**	**0.882**	**0.821**
**Trained with only internal set (Cohort 1 Train), tested on external set (Cohort 4 Test)**							
**Subtype**	**Google**	**UNI-v2**	**CONCH-v1.5**	**TITAN**	**Virchow**	**PRISM**	**ResNet50**
Dermatofibrosarcoma protuberans	0.892	0.892	0.784	0.946	0.919	0.892	0.703
Neurofibroma	0.795	0.974	0.872	0.897	0.897	0.641	0.051
Nodular fasciitis	0.412	0.706	0.471	0.824	0.765	0.647	0.000
Desmoid fibromatosis	0.706	0.765	0.265	0.882	0.853	0.882	0.029
Synovial sarcoma	0.654	0.728	0.704	0.753	0.778	0.802	0.407
Lymphoma	0.795	0.932	0.786	0.889	0.863	0.915	0.573
Glomus tumour	1.000	1.000	1.000	1.000	1.000	1.000	1.000
Intramuscular myxoma	0.576	0.273	0.273	0.788	0.394	0.606	0.424
Ewing	0.615	1.000	0.846	0.692	1.000	0.923	0.385
Schwannoma	0.688	0.922	0.922	0.896	0.909	0.883	0.494
Myxoid liposarcoma	0.795	0.923	0.897	0.974	0.974	0.923	0.256
Leiomyosarcoma	0.875	0.922	0.859	0.938	0.938	0.938	0.672
Solitary fibrous tumour	0.607	0.832	0.842	0.816	0.842	0.633	0.709
Low grade fibromyxoid sarcoma	0.889	0.921	0.794	0.873	0.889	0.667	0.825
**Overall accuracy**	**0.721**	**0.847**	**0.772**	**0.860**	**0.852**	**0.782**	**0.533**
**Trained with internal and external sets (Cohort 1 Train and Cohort 4 Train), tested on internal set (Cohort 1 Test)**							
**Subtype**	**Google**	**UNI-v2**	**CONCH-v1.5**	**TITAN**	**Virchow**	**PRISM**	**ResNet50**
Dermatofibrosarcoma protuberans	0.850	0.850	0.900	0.950	0.950	0.850	0.750

Neurofibroma	0.700	1.000	0.900	0.700	1.000	0.800	0.700
Nodular fasciitis	0.900	0.900	0.800	0.950	0.944	0.850	0.800
Desmoid Fibromatosis	0.900	0.800	0.850	0.900	0.900	0.900	0.800
Synovial sarcoma	0.950	0.950	0.800	0.900	0.950	0.950	0.900
Lymphoma	0.900	1.000	1.000	1.000	1.000	0.950	0.900
Glomus tumour	1.000	1.000	1.000	1.000	1.000	1.000	0.900
Intramuscular myxoma	1.000	1.000	1.000	1.000	0.950	1.000	0.900
Ewing	0.950	0.950	0.950	1.000	1.000	1.000	0.800
Schwannoma	0.900	0.900	0.850	0.900	0.900	0.900	0.800
Myxoid liposarcoma	0.850	0.900	0.800	1.000	0.850	0.800	0.800
Leiomyosarcoma	0.800	0.800	0.850	0.850	0.850	0.750	0.900
Solitary fibrous tumour	0.900	0.900	0.950	0.950	0.900	0.800	0.850
Low grade fibromyxoid sarcoma	1.000	0.900	1.000	0.950	1.000	0.850	0.950
**Overall**	**0.900**	**0.918**	**0.904**	**0.932**	**0.942**	**0.886**	**0.839**
**Trained with internal and external sets (Cohort 1 Train and Cohort 4 Train), tested on external set (Cohort 4 Test)**							
**Subtype**	**Google**	**UNI-v2**	**CONCH-v1.5**	**TITAN**	**Virchow**	**PRISM**	**ResNet50**
Dermatofibrosarcoma protuberans	0.946	0.973	0.811	0.973	0.919	0.838	0.757
Neurofibroma	0.872	0.974	0.897	0.897	0.923	0.795	0.667
Nodular fasciitis	0.706	0.882	0.529	0.882	0.765	0.765	0.235
Desmoid Fibromatosis	0.824	0.882	0.824	0.912	0.882	0.912	0.618
Synovial sarcoma	0.654	0.790	0.654	0.790	0.753	0.778	0.630
Lymphoma	0.897	0.923	0.915	0.932	0.983	0.906	0.923
Glomus tumour	1.000	1.000	1.000	1.000	1.000	1.000	1.000
Intramuscular myxoma	0.970	0.515	0.788	0.879	0.697	0.788	0.697
Ewing	0.769	1.000	0.923	0.846	0.923	0.923	0.538
Schwannoma	0.792	0.935	0.909	0.883	0.779	0.870	0.740
Myxoid liposarcoma	0.872	0.974	0.949	1.000	0.923	0.923	0.846
Leiomyosarcoma	0.891	0.969	0.906	0.938	0.922	0.938	0.797
Solitary fibrous tumour	0.704	0.918	0.842	0.827	0.842	0.658	0.709
Low grade fibromyxoid sarcoma	0.873	0.873	0.762	0.857	0.889	0.714	0.746
**Overall**	**0.808**	**0.899**	**0.838**	**0.881**	**0.865**	**0.803**	**0.736**
